# Immobilization of Laccase on Magnetic Chelator Nanoparticles for Apple Juice Clarification in Magnetically Stabilized Fluidized Bed

**DOI:** 10.3389/fbioe.2020.00589

**Published:** 2020-07-02

**Authors:** Feng Wang, Mariam Owusu-Fordjour, Ling Xu, Zhongyang Ding, Zhenghua Gu

**Affiliations:** ^1^School of Food and Biological Engineering, Jiangsu University, Zhenjiang, China; ^2^Institute of Food Physical Processing, Jiangsu University, Zhenjiang, China; ^3^National Engineering Laboratory for Cereal Fermentation Technology, Jiangnan University, Wuxi, China; ^4^Jiangsu Provincial Research Center for Bioactive Product Processing Technology, Jiangnan University, Wuxi, China

**Keywords:** laccase immobilization, magnetic nanoparticles, juice clarification, alternating magnetic field, magnetically stabilized fluidized bed

## Abstract

The juice clarification, one of the key steps in juice processing, suffers from haze formation that results from residual phenolic compounds. In this study, laccase was immobilized on metal-chelated magnetic silica nanoparticles and used for continuous juice clarification in a magnetically stabilized fluidized bed (MSFB) assisted by alternating magnetic field. Furthermore, a new combination of laccase catalysis and microfiltration was developed for the juice clarification. Immobilized laccase provided high relative activity within broader ranges of pH and temperature compared to the free enzyme. Magnetic immobilized laccase exhibited the best reaction rate of 12.1 μmol g^–1^ min^–1^ for catechol oxidation under the alternating magnetic field of 400 Hz, 60 Gs. No activity loss occurred in immobilized laccase after 20 h continuous operation of juice treatment in MSFB under an alternating magnetic field. Combined with microfiltration after treatment with immobilized laccase, the color of apple juice was decreased by 33.7%, and the light transmittance was enhanced by 20.2%. Furthermore, only 16.3% of phenolic compounds and 15.1% of antioxidant activity was reduced for apple juice after the clarification. By this combination strategy, the apple juice possessed good freeze–thaw and thermal stability.

## Introduction

The consumption of fruit juice continuously increases due to the well-known beneficial effects of fruit juice and the consumers’ continued pursuit of health and wellness (Bharate and Bharate, 2014; Kahraman et al., 2017). There are a lot of bioactive compounds in apple juice, including flavonoids, carotenoids, polyphenols, and vitamins (Belgheisi and Kenari, 2019). Among these compounds, polyphenols are a natural source associated with antioxidant activity and health benefits (Agcam et al., 2014). However, polyphenols can react with protein in fruit juice and cause a maderization process during storage, which leads turbidity, intensified color, unpleasant taste, haze, and sediments (Ozdal et al., 2013; Pezzella et al., 2015). In fruit juice, there are two main mechanisms in color formation, including non-enzymatic and enzymatic browning. Besides the Maillard reaction of non-enzymatic browning, enzymatic oxidation also plays an important role in the browning process of apple juice, where polyphenoloxidase can oxidize reactive phenolic compounds into brown pigments precursors-quinones (Paravisini and Peterson, 2018; Martínez-Hernández et al., 2019). The increase in color intensity in apple juice exhibited a negative effect on consumer acceptance (Paravisini and Peterson, 2018). Different clarification methods have been applied in the fruit juice industry, such as thermal treatment, absorbents/flocculants, centrifugation, and/or filtration (Lettera et al., 2016; Belgheisi and Kenari, 2019). Although these strategies can improve the clarity and stability of fruit juice, the formation of haze cannot be avoided due to the presence of reactive phenolic compounds (Lettera et al., 2016).

Enzymatic methods are also important and useful for the clarification of fruit juice. Pectinase one of the important enzymes in fruit juice clarification technology, and it can catalyze the degradation of pectic substances and improve the clarity, extraction yield, and filterability of juice (de Oliveira et al., 2018; Deng et al., 2019). Besides pectinase, endo-β-mannanase, endo-β-xylanase, β-glucosidase, β-xylosidase, α-galactosidase, and protease have also been investigated in terms of juice clarification (Benucci et al., 2019; Suryawanshi et al., 2019). However, these enzymes cannot catalyze the reaction with polyphenol and remove these phenolic compounds. Laccases are multicopper oxidoreductase enzymes that can oxidize monophenols and/or polyphenols into polymers with the reduction of molecular oxygen to water (Wang et al., 2019). Due to this useful reaction, laccase has attracted research interests and been applied in juice clarification to remove the polyphenols by polymerization (Bezerra et al., 2015; Lettera et al., 2016).

Immobilization of enzymes provides an excellent tool for their industrial application by improving the stability of enzyme activity and the tolerance to environmental condition and inhibitors and affording good reusability (Deng et al., 2019). Among different supports, magnetic nanoparticles are good candidates for enzyme immobilization due to superparamagnetism, low toxicity, and the ease with which they separate (Ou et al., 2019). With the assistance of an alternating magnetic field, magnetic supports can oscillate in the direction of a magnetic field and behave like microscopic stirrers, which can increase mass transfer in enzymatic reaction and result in higher catalytic efficiency for magnetic immobilized enzymes (Liu et al., 2015; Xia et al., 2018). To carry out the continuous operation, a magnetically stabilized fluidized bed (MSFB) has been used in wastewater treatment and chiral compounds synthesis with enzymes immobilized on magnetic supports (Wang et al., 2012; Ou et al., 2019). Among different immobilization technologies, metal-chelated adsorption of enzymes on metal-chelated adsorbents belongs to reversible non-covalent immobilization, resulting in strong absorption and good reusability of supports (Wang et al., 2008). Due to the high quantity and accessibility of His residues on the surface of the *Trametes versicolor* laccase protein, Cu^2+^-chelated support was regarded as a good candidate and has been previously utilized for laccase immobilization (Wang et al., 2010).

In the process of laccase-catalyzed juice clarification, a significant increase in color and turbidity occurred in treated juice due to the formation of soluble oligomers (Neifar et al., 2011; Bezerra et al., 2015). To remove the polymers after laccase treatment, ultrafiltration was generally used and caused a sharp decrease in the phenolic compound and antioxidant activity for juice (Neifar et al., 2011). Therefore, there is still an interest in exploring a laccase-catalyzed clarification method with less loss of antioxidant activity and better reduction in color and turbidity. In this study, laccase from *T. versicolor* immobilized Cu^2+^-chelated magnetic silica nanoparticles, and the catalytic characteristics and stability of immobilized laccase was investigated. An alternating magnetic field was applied in the clarification of apple juice with the immobilized laccase, and a combination of laccase catalysis and microfiltration was established for juice clarification with high quality. In addition, a continuous operation of juice clarification in MSFB assisted by alternating magnetic field was investigated.

## Materials and Methods

### Materials

Laccase from *T. versicolor* (powder, ≥0.5 U/mg), 3-chloropropyltrimethoxysilane (CPTS, ≥97%), and 1,1-diphenyl-2-picrylhydrazyl radical (DPPH, 95%) were purchased from Sigma-Aldrich (St. Louis, MO, United States). All other materials are of analytical grade and were provided by Sinopharm Chemical Reagent Co., Ltd. (Shanghai, China). All materials were used as received without any further purification.

### Preparation of Magnetic Silica Nanoparticles and Surface Modification

Magnetite nanoparticles of Fe_3_O_4_ were synthesized by a chemical precipitation method (Wang et al., 2010). Briefly, 0.99 g of FeCl_2_⋅4H_2_O and 2.7 g FeCl_3_⋅6H_2_O were dissolved in 100 mL of de-aerated millipore water under a N_2_ atmosphere. After heated to 80°C, 10 mL NH_4_OH was added to the solution and the reaction was kept for 30 min at 80°C under constant stirring. The obtained Fe_3_O_4_ particles were collected, washed with millipore water and dispersed in millipore water for further use.

Magnetic silica nanoparticles were fabricated by the modified method reported by Deng et al. (2005). Briefly, 160 mL of isopropanol, 40 mL millipore water, and 5 mL ammonia aqueous were mixed under vigorous stirring, and the desired amount of magnetite nanoparticles (Fe_3_O_4_) was added to the mixture. Finally, 1 mL of tetraethylorthosilicate (TEOS) was slowly added to this dispersion under stirring. After 12 h stirring, silica was formed on the surface of magnetite nanoparticles through hydrolysis and condensation of TEOS. The resulted magnetic silica nanoparticles were collected, washed with millipore water and dispersed in millipore water for further use. The magnetic silica nanoparticles possessed spherical shape with an average diameter of 213 nm (Supplementary Figure S1).

Surface modification of magnetic silica nanoparticles was conducted according to the method described by Wang et al. (2010). In this method, 200 mg of magnetic silica nanoparticles was dispersed in the mixture of 20 mL water and 80 mL ethanol. Two milliliters of CPTS were added to the dispersion and sonicated for 30 min at pH 4.5. The resulted particles were collected by magnet and dispersed in 100 mL, pH 8.5 NaOH solution containing 6 g of iminodiacetic acid (IDA) followed by stirring at 60°C for 10 h under nitrogen gas atmosphere. Particles were recovered, washed with millipore water and stirred in 100 mg mL^–1^ of CuSO_4_ solution for 1 h. The final obtained Cu^2+^-chelated magnetic silica nanoparticles were washed, dispersed in water and used as supports for laccase immobilization.

### Laccase Immobilization

Laccase adsorption on Cu^2+^-chelated magnetic silica nanoparticles was tested in tartaric acid buffer of pH 3.0. For this purpose, 50 mg of magnetic particles was added into 25 mL of laccase solution with the concentration of 0.05–0.3 mg mL^–1^ prepared in the buffer. The resulting suspensions were subsequently incubated at 25°C at 150 rpm for 1 h in order to reach adsorption equilibrium. The laccase-adsorbed particles were separated by magnet and washed with the same buffer until no protein was detected in the supernatant. The elution solution containing residual laccase was collected. The activities of the immobilized laccase were evaluated by the assay of the activity recovery. The activity recovery of the immobilized laccase was calculated from Equation (1):

(1) $$R\left(\%\right)=\left(\frac{A_{i}}{A_{f}}\right)\times 100\%$$

where, *R* is the activity recovery of the immobilized laccase (%), *A*_*i*_ the activity of the immobilized laccase (U), and *A*_*f*_ is the activity of the same amount of free laccase in solution as that immobilized on particles (U).

The amount of protein in liquid solution was determined by the Bradford method (Bradford, 1976), and the amount of protein bound on the particles and the immobilization efficiency were calculated from Equation (2).

(2) $$IE\left(\%\right)=\left(\frac{P_{i}}{P_{T}}\right)\times 100\%$$

where, *IE* is the immobilization efficiency of laccase on magnetic supports (%), *P*_*i*_ the laccase protein immobilized on magnetic supports (mg), and *P*_*T*_ is the total laccase protein added in reaction solution (mg).

The maximal activity recovery of 62.1% and the best immobilization efficiency of 98.6% were achieved at the laccase concentration of 0.1 mg mL^–1^ (Supplementary Figure S2). Under this condition, the adsorbed laccase on Cu^2+^-chelated magnetic silica nanoparticles 49.3 mg g^–1^-particles, which performed the laccase activity of 15.3 U g^–1^-particles. The resulting immobilized laccase were stored at 4°C in fresh buffer until use.

### Determination of Laccase Activity

A laccase activity array of the free and immobilized laccase was carried out according to the spectrophotometrical method (Wang et al., 2010). In this test, 0.1% (w/v) catechol in 100 mM tartaric acid buffer (pH 3.0) was used as substrate for the oxidation by laccase at 25°C. A suitable amount of laccase was added to the substrate solution, and the increase in the absorbance of supernatant was determined at 450 nm in a UV-2100 spectrophotometer (Unico Company, Shanghai, China). The molar absorption coefficient of catechol is 2211 M^−1^⋅cm^–1^. One unit of activity is defined as the amount of laccase required to oxidize 1 μmol of catechol per minute. The effects of pH and temperature on free and immobilized laccase activity were tested as the relative activity under a variety of pH values (pH 2.5–7.0) and temperatures (20–80°C).

### Array of Kinetic Parameters and Stability of Free and Immobilized Laccase

Kinetic parameters of the Michaelis–Menten equation (K_m_ and *k*_cat_) for free and immobilized laccase were determined by measuring initial rates of the reaction with catechol (0.05∼10 mM) in buffer solutions at 25°C. The kinetic constants were obtained by fitting the data to the Michaelis–Menten equation using a non-linear regression code (Origin 2019b).

Thermal stabilities of the free and immobilized laccase were tested by measuring the residual laccase activity after they were exposed to 60°C in sodium acetate buffer (0.1 M, pH 4.0). Storage stabilities of free and immobilized laccase were determined by measuring the residual laccase activity after storage in a sodium acetate buffer (0.1 M, pH 4.0) for 10 weeks.

### Oxidation of Catechol by Immobilized Laccase Under Alternating Magnetic Field

**SCHEME 1:**
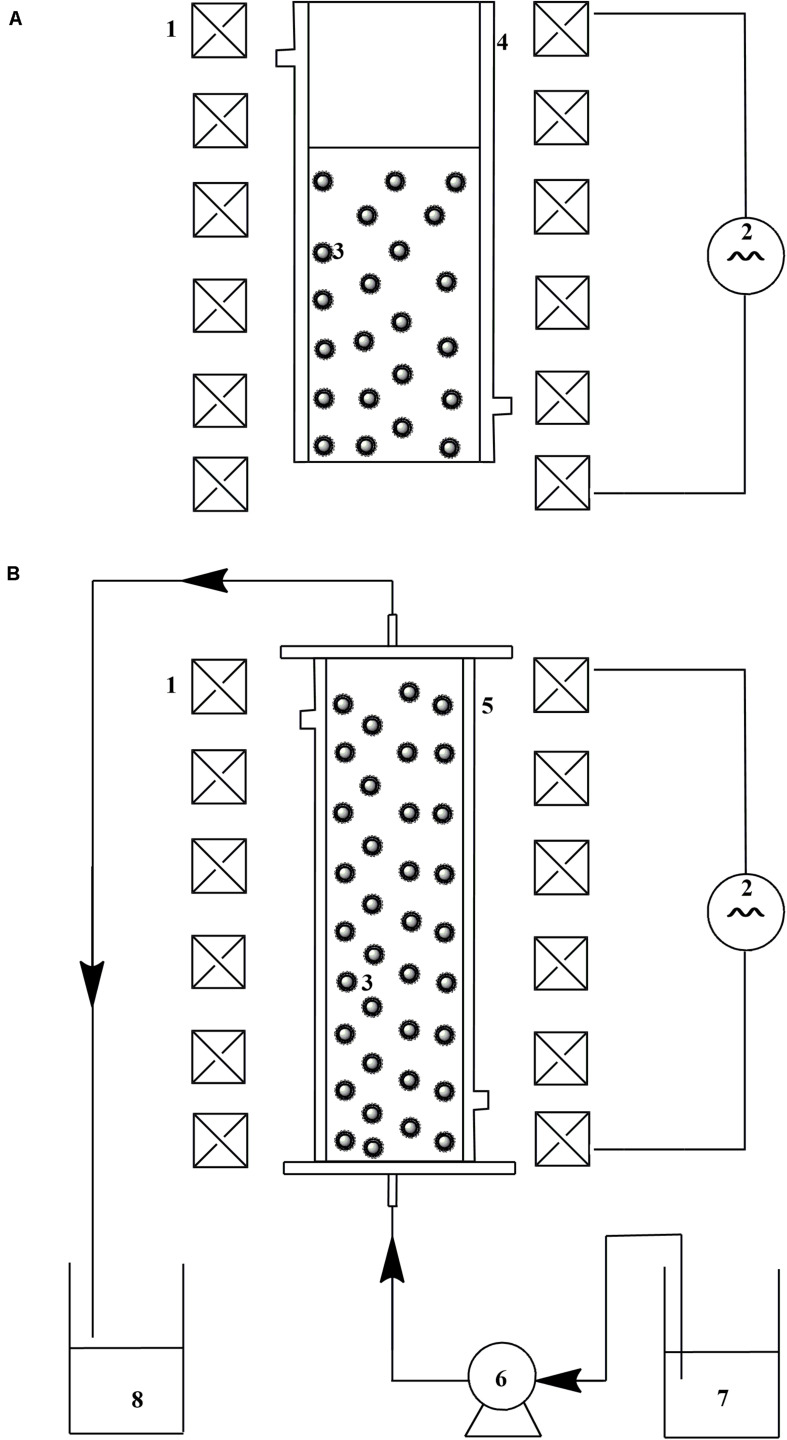
Schematic diagram of device for catalysis with magnetic immobilized enzyme assisted by alternating magnetic field: **(A)** reactor for batch treatment; **(B)** MSFB for continuous treatment. 1. Helmholtz coil; 2. AC power supply; 3. magnetic immobilized laccase; 4. glass column for batch treatment; 5. glass column for fluidized bed; 6. peristaltic pump; 7. untreated apple juice; 8. treated apple juice.

The reaction rate of catechol oxidation catalyzed by immobilized laccase was determined according to the method described by Xia et al. (2018). To carry out this determination, the desired amount of immobilized laccase was added to 10 mM catechol dissolved in sodium acetate buffer (0.1 M, pH 5.0) under an alternating magnetic field at 25°C. A reaction under static condition and a reaction under mechanical stirring were set as the controls, and the stirring speed was optimized and 150 rpm selected as the optimal condition. During the catalysis process, the sample was collected with a certain time interval and the absorbance variation of supernatant was determined at 450 nm in a UV-2100 spectrophotometer. A lab-designed device was used for the oxidation of catechol by immobilized laccase under alternating magnetic field (Scheme 1A). A set of Helmholtz coils connected to AC power supply were used to produce the axially alternating magnetic field (frequency: 50–600 Hz, field strength: 20–200 Gs). A glass column (D_in_: 4.0 cm) surrounded by a water jacket was used as the reactor for the catalysis. The reaction solution possessed a volume of 100 mL and its temperature was maintained at 25 ± 1°C by circulating water through the water jacket.

### Fruit Juice Treatment by Immobilized Laccase

Apples were purchased from a local supermarket (Zhenjiang, China), and the apple juice was prepared based on the method describe by Bezerra et al. (2015). Briefly, the apples were rinsed and triturated, and the juice was pressed through four layers of gauze. Kaolin of 0.1 mg mL^–1^ was added to the juice. The mixture was shaken at 50 rpm at 25°C for 30 min and then centrifuged at 4000 *g* at 4°C for 30 min. The resulted supernatant was used as apple juice. To determine the effect of immobilized laccase dosage on juice clarification, the enzymatic treatment of apple juice was conducted in the device shown in Scheme 1A with different concentrations of immobilized laccase (0.2–2 g L^–1^) at 35°C for 0.5 h. The operation temperature of the immobilized laccase was selected based on the consideration of its relative activity and stability for long-time operation. The optimal frequency and field strength obtained in the above section was applied for the batch operation. The treated apple juice was filtrated with Amicon^®^ Ultra centrifugal ultrafiltration tube (10 kDa). For the test of the combination with microfiltration, the treated apple juice was filtrated with a microporous membrane filter of 0.45 μm.

The reusability of the immobilized laccase was also assessed in the clarification of apple juice. Several consecutive operating cycles were performed by juice treatment. At the end of each treatment, the immobilized laccase was washed three times with water and the procedure was repeated with a fresh aliquot of apple juice. The residual laccase activity was detected after each cycle.

Continuous treatment of apple juice by immobilized laccase was tested in the MSFB assisted by alternating magnetic field (Scheme 1B). The same generator of magnetic field was used as described in Scheme 1A. A glass column (D_in_: 2.5 cm, H: 40 cm) with a water jacket was used for the enzyme catalysis in the fluidized bed. The treatment temperature was maintained at 35 ± 1°C by circulating water through the water jacket. The optimal frequency and field strength obtained in the above section was applied for the continuous operation. Apple juice was pumped into the glass column by way of a peristaltic pump, and the residence time was controlled at 0.5 h. The concentration of immobilized laccase in working volume was 1 g L^–1^.

For the analysis of polymer size in apple juice treated by laccase, the batch treatment of apple juice was carried out with immobilized laccase at the optimal condition under alternating magnetic field. The treatment of apple juice with immobilized laccase was also conducted without a magnetic field, and mechanical stirring of 150 rpm was applied. The treatment of apple juice with free enzyme was tested with the same laccase activity of 15.3 U L^–1^ at 150 rpm, 35°C for 0.5 h. After laccase treatment, the size of generated polymer in apple juice was analyzed by DelsaNano C Particle Size and Zeta Potential Analyzer (Beckman Coulter Inc., Brea, CA, United States).

### Characterization of Apple Juice Before and After Enzymatic Treatment

The transmittance of apple juice was spectrometrically measured at 650 nm with distilled water as a reference (Deng et al., 2019). The color value of apple juice was spectrometrically measured at 430 nm with distilled water as a reference after the mixture of juice sample with equal volume of ethanol for 30.0 min (Deng et al., 2019). Total phenolics in apple juice were determined with Folin-Ciocalteau reagent using a gallic acid standard (Lettera et al., 2016). The antioxidant activity of apple juice was determined with chromogen radical DPPH test (Wu et al., 2003).

### Stability Array of Apple Juice

Apple juice before and after treatment was heated at 70°C for 30 min and froze at −20°C for 24 h, respectively. The light transmittance of apple juice before and after treatment was compared after processing to determine the stability of the juice.

### Statistical Analysis

All experiments were completed in triplicate. Experimental results were expressed as mean ± standard deviation. Significances of comparisons between different data were conducted by SPSS version 22 (SPSS Inc., Chicago, IL, United States). Independent-sample *T* tests and a one-way variance were applied.

## Results and Discussion

### Property of Immobilized Enzymes

**FIGURE 1:**
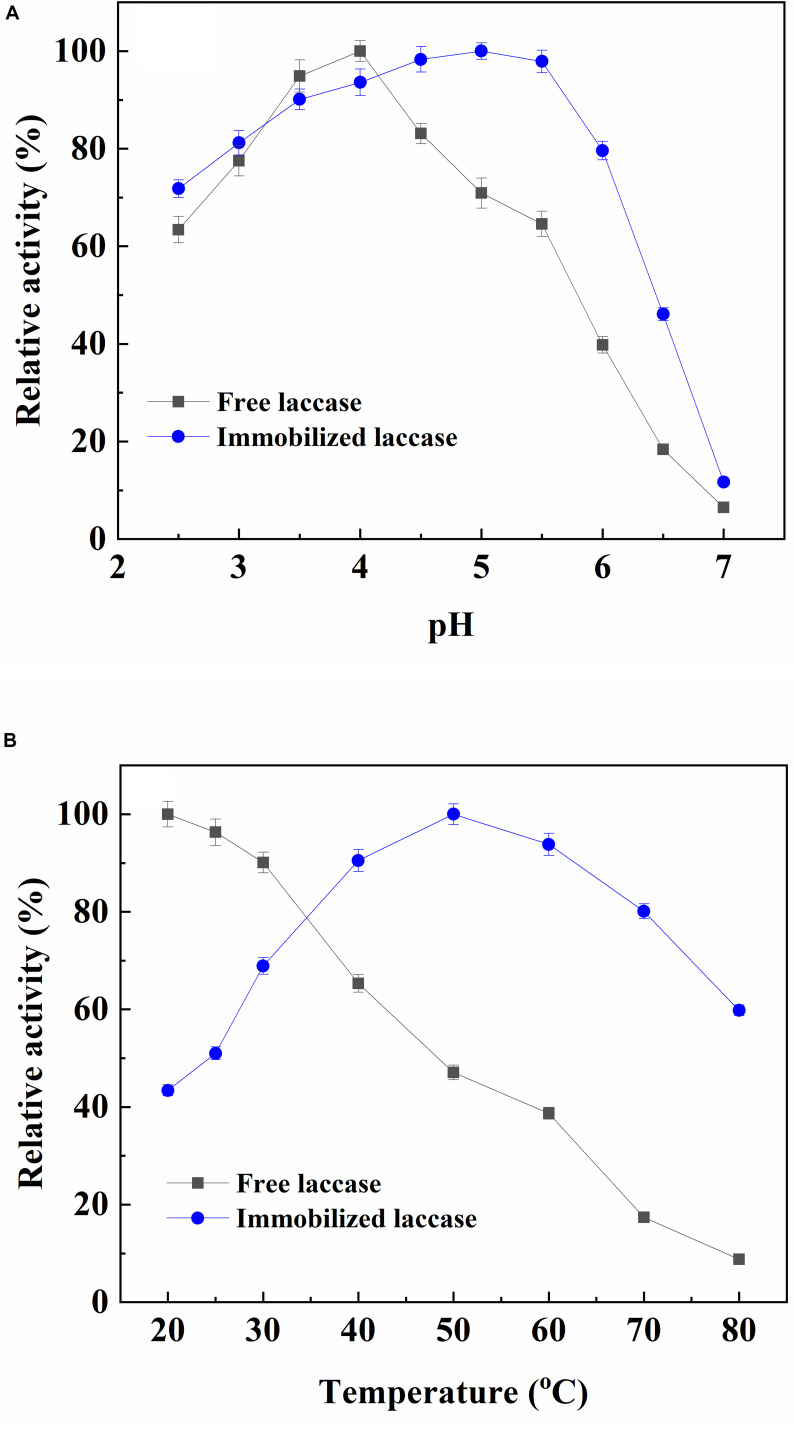
Effect of pH **(A)** and temperature **(B)** on the activity of free and immobilized laccase.

The optimum pH for the free laccase was 4.0, and the immobilized enzyme exhibited the highest activity with an interval in 4.5–5.5 (Figure 1A). The slight shift of the optimum pH after laccase immobilization could be resulted from the ionic interaction between charged surface of magnetic support and laccase (Yavuz et al., 2002). These results also indicated that the immobilized laccase can be more stable within a wider pH range. The optimal temperature of free and immobilized laccase was 20°C and 50°C, respectively (Figure 1B). The optimal temperature of the adsorbed laccase performed a shift toward high temperature. It was due to the increased activation energy of immobilized laccase resulted from multipoint chelate interactions that caused laccase to reorganize an optimum conformation for substrate binding at a high temperature (Kara et al., 2005; Sari et al., 2006). The activity of free laccase decreased with the increasing temperature, while the immobilized laccase maintained more than 90% of its highest activity with a broader profile of 40–60°C. This phenomenon was because of the restricted conformational mobility of the immobilized proteins (Yodoya et al., 2003; Wang et al., 2010).

The apparent K_m_ value of the immobilized laccase was 3.17 mM, which was much higher than that of the free enzyme (1.22 mM) (Supplementary Table S1). The reduced affinity of adsorbed laccase to its substrate was probably due to structural change in the immobilization process, resulting in low accessibility of the substrate to enzyme active site (Bai et al., 2006; Sari et al., 2006). No significant difference occurred in the catalytic constant (*k*_cat_) for free and immobilized laccase. However, the ratio of *k*_cat_/K_m_ to the immobilized enzyme was lower than the value of free one (Supplementary Table S1). A similar phenomenon was also observed in laccase immobilization on the other carriers (Rekuc et al., 2008; Wang et al., 2010).

After 4 h incubation at 60°C, 15.7% and 65.2% of their initial activity were maintained for free and immobilized laccase, respectively (Supplementary Figure S3A). The immobilized laccase performed better thermal stability than free laccase. In the test of storage stability, no activity was detected for free laccase after 8 weeks of storage, whereas the immobilized laccase possessed 95.1% of its initial activity after 10 weeks of storage (Supplementary Figure S3B). Therefore, the immobilization of laccase on Cu^2+^-chelated magnetic silica nanoparticles was beneficial for the improvement of laccase stability, where enzyme active conformation could be maintained by multipoint bond interaction between carrier and enzyme (Sari et al., 2006).

### Catechol Oxidation by Immobilized Laccase Under Different Alternating Magnetic Fields

**FIGURE 2:**
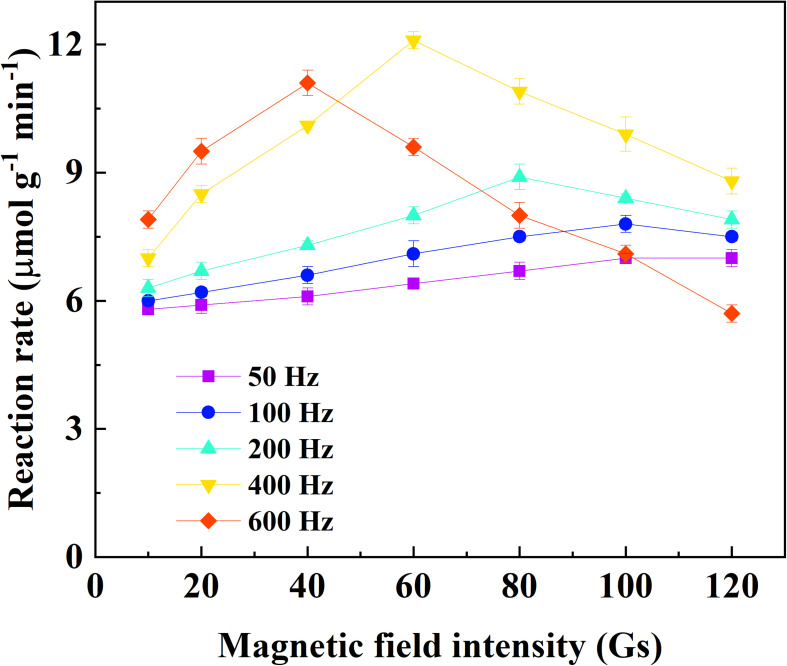
Effect of frequency and field intensity on the reaction rate of catechol oxidation catalyzed by immobilized laccase under alternating magnetic field.

**FIGURE 3:**
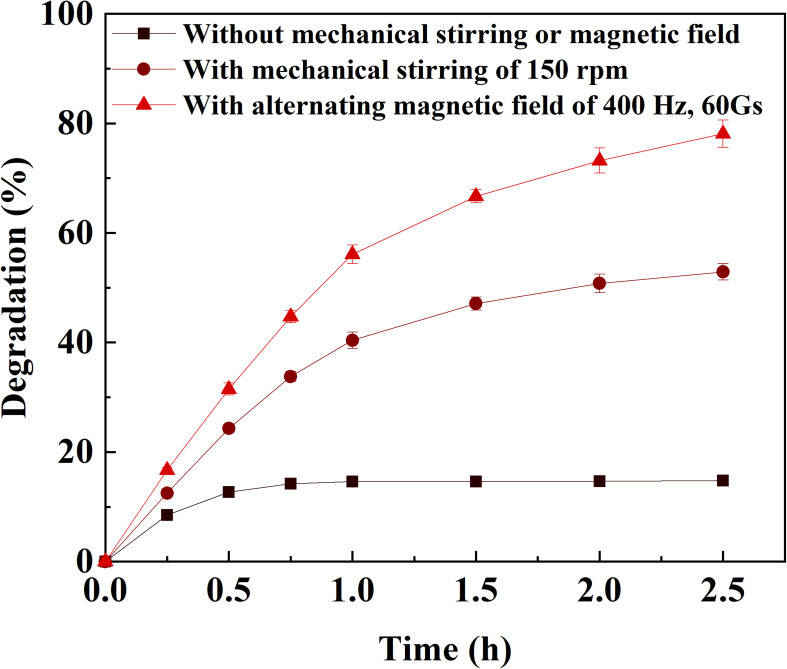
Catechol oxidation catalyzed by immobilized laccase under different conditions.

The effects of frequency and field strength on the reaction rate of catechol oxidation by immobilized laccase was investigated with the assistance of alternating magnetic field (Figure 2). At a fixed frequency, the reaction rate increased to the peak with respect to the field strength and then declined if the field strength continued increasing. It indicated that there was a compromise between frequency and field strength for the catechol oxidation catalyzed by immobilized laccase. The highest reaction rate of 12.1 μmol g^–1^ min^–1^ was achieved under the alternating magnetic field of 400 Hz and 60 Gs. Under the field strength of no more than 40 Gs, the reaction rate was enhanced when magnetic field of higher frequency was applied. This is consistent with the results reported by Liu et al. (2015). The magnetic immobilized laccase performed as nano-stirrers and more vigorous particle motion was achieved under higher field strength, resulting in better mass transfer (Liu et al., 2015; Xia et al., 2018). In addition, rapid change of magnetic field direction under high frequency could force the motion of magnetic particle along with the magnetic field and protect from the formation of aligning particles (Liu et al., 2015). However, the oscillating and/or stirring resulting from the alternating magnetic field may generate too much shear stress for the enzyme on magnetic support under higher frequency and field strength, which can cause deactivation of the enzyme. This has also been proven by the reported results (Ou et al., 2019). Compared to the degradation of catechol under static or mechanical-stirring condition, the reaction rate and degradation efficiency of catechol catalyzed by the immobilized laccase was greatly improved by the application of alternating magnetic field (Figure 3). The initial reaction rate of catechol oxidation was 6.2 μmol g^–1^ min^–1^ and 9.1 μmol g^–1^ min^–1^ for static condition and mechanical-stirring, respectively. The alternating magnetic field did not affect the activity of free laccase (data not shown). Based on the above results, the alternating magnetic field of 400 Hz, 60 Gs was selected as optimal condition for further treatment of apple juice by the immobilized laccase.

### Batch Treatment of Apple Juice With Immobilized Laccase

**FIGURE 4:**
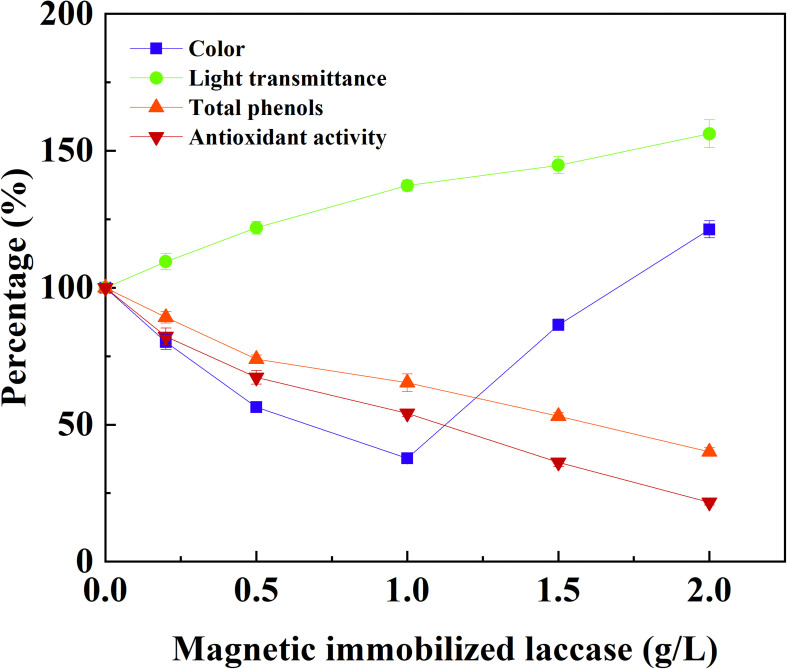
Effect of concentration of magnetic immobilized laccase on the treatment of apple juice under an alternating magnetic field.

**FIGURE 5:**
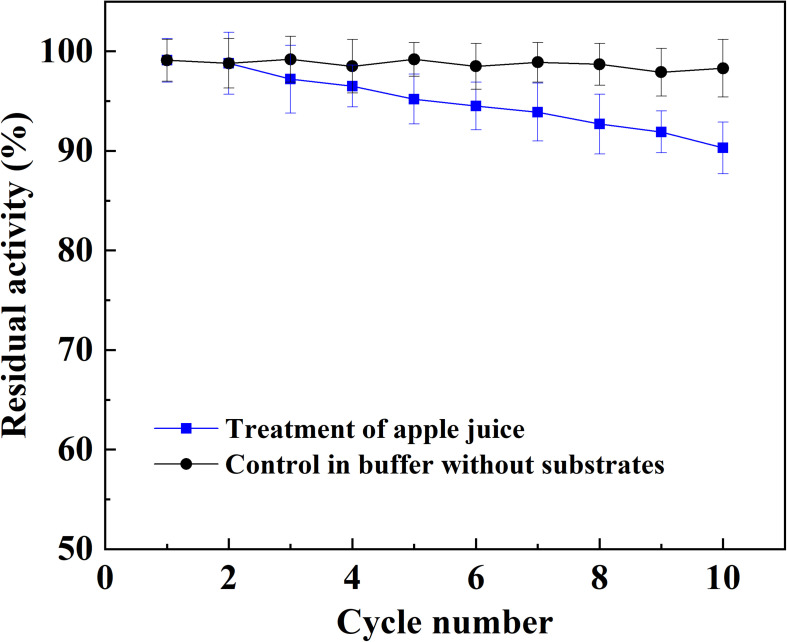
Reusability of magnetic immobilized laccase in the batch treatment of apple juice.

The application potential of magnetic immobilized laccase in fruit juice clarification was evaluated using apple juice as a model system. The treatment of apple juice with different dosage of magnetic immobilized laccase was conducted for 0.5 h under an alternating magnetic field, and this was followed by ultrafiltration. Total phenolic compounds and antioxidant activity decreased with the increased concentration of immobilized laccase, and the light transmittance was improved at the same time (Figure 4). More immobilized laccase was beneficial for the reduction of turbidity, which caused the increased loss of antioxidant activity. For the color of apple juice, the minimum was obtained at 1 g L^–1^ of magnetic immobilized laccase, and the color increased sharply when more immobilized laccase was added. This may be due to oxidation of a more phenolic compound in low molecular weight besides polyphenols and production of more soluble oligomers (Neifar et al., 2011). In this study, the phenolic compounds, including ferulic acid, phloridzin, phloretin, epicatechin, caffeic acid, rutin, catechin, and chlorogenic acid, were presented in apple juice. In addition, catechin and chlorogenic acid were oxidized more easily, and an increased laccase dosage resulted in more oxidation of other phenolic compounds (data not shown). Therefore, 1 g L^–1^ of magnetic immobilized laccase was selected for the optimum concentration for the treatment of apple juice where the transmittance was increased by 37.3%, and the total phenolic compounds and antioxidant activity were decreased by 34.7% and 45.9%, respectively. High phenolic reduction in apple juice also occurred in other reported treatments with immobilized laccase (Bezerra et al., 2015; Lettera et al., 2016). The magnetic catalyst remained 90.3% of its initial activity after reusing for 10 batches, exhibiting good reusability in juice treatment (Figure 5).

### Effect of Microfiltration on Treatment of Apple Juice With Immobilized Laccase

**FIGURE 6:**
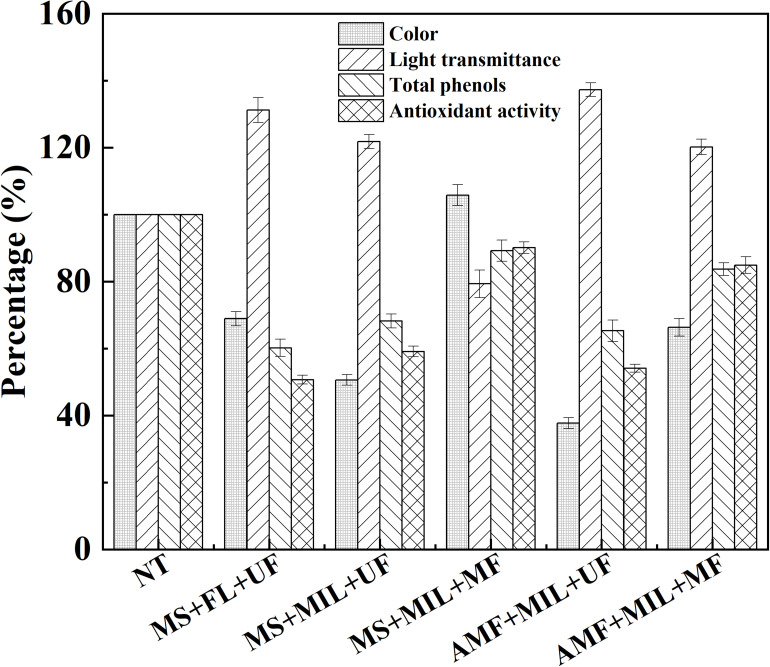
Treatments of apple juice with magnetic immobilized laccase under different condition. NT, not treated; MS, mechanical stirring; FL, free laccase; UF, ultrafiltration; MIL, magnetic immobilized laccase; MF, microfiltration; AMF, alternating magnetic field.

Due to the loss of phenolic compounds and antioxidant activity during the filtration process, the application of microfiltration was investigated after the treatment of apple juice with immobilized laccase. With the help of ultrafiltration, treatment under an alternating magnetic field provided the best indexes compared to those under mechanical stirring (Figure 6). In the case of microfiltration combined with magnetic immobilized laccase, enhanced color and turbidity occurred in apple juice after the treatment by mechanical stirring. However, in the treatment under alternating magnetic field, the color was reduced by 33.7%, and the light transmittance was increased by 20.2%, where only 16.3% of total phenolic compound and 15.1% of antioxidant activity were lost after microfiltration. Therefore, the treatment of apple juice with immobilized laccase under alternating magnetic field can be combined with microfiltration for the juice clarification. By this strategy, the color and light transmittance were improved, and more phenolic compounds remained. Compared to ultrafiltration, microfiltration possessed better energy saving and lower operation pressure in juice treatment, which is beneficial for its industrial application (Ilame and Singh, 2015). To illustrate the possible reason for this phenomenon, the size distribution of polymers after the treatment with laccase was determined before filtration (Supplementary Figure S4). The smallest polymer diameter of 291.6 nm and the broadest size distribution was obtained in the treatment with free laccase under mechanical stirring. Thus, the apple juice after the treatment with free laccase under mechanical stirring exhibited the highest color compared to the other treatments followed by the ultrafiltration (Figure 6). The average diameters of polymers in apple juice after the treatment of immobilized laccase were 611.6 nm and 1326.5 nm for mechanical stirring and alternating magnetic field, respectively. In addition, the treatment under alternating magnetic field performed the narrowest size distribution of polymer. It indicated the mixing by nano-stirring and magnetic oscillating can also facilitate the formation of larger polymers, making microfiltration combination possible in the treatment of apple juice with magnetic immobilized laccase.

### Continuous Treatment of Apple Juice With Immobilized Laccase in MSFB

**FIGURE 7:**
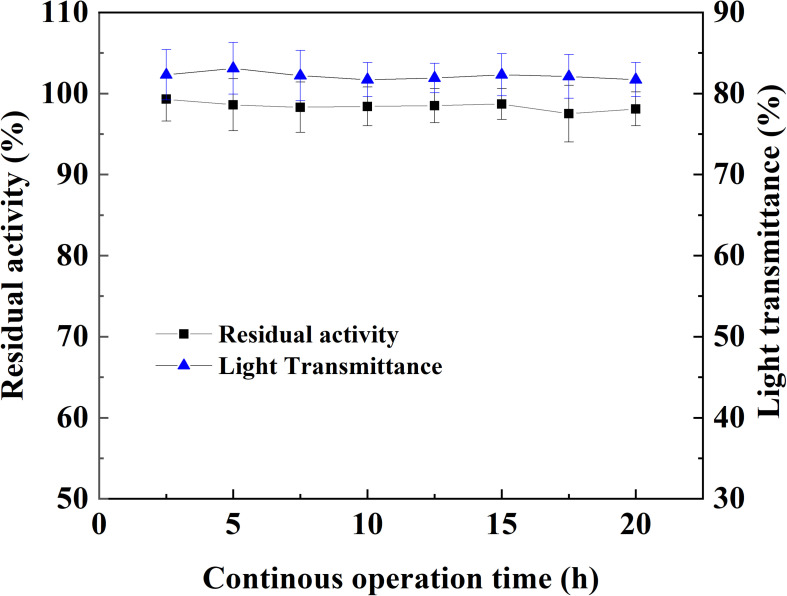
Operation stability of magnetic immobilized laccase in the continuous treatment of apple juice with MSFB assisting by an alternating magnetic field.

The operation stability of magnetic immobilized laccase in continuous treatment of apple juice was tested in MSFB assisted by an alternating magnetic field where microfiltration was used after the enzyme treatment. No significant variety happened in the light transmittance and the magnetic catalyst maintained its initial enzyme activity after 20 h treatment (Figure 7). Even when the treatment of apple juice was prolonged to 100 h, there was only 5.3% activity lost occurred in the immobilized laccase. The continuous operation in MSFB was beneficial for the stability of magnetic catalyst. The flowing substrate could provide quick and complete removal of oxidation products and reduce the inhibitory effect resulted from polymers (Wang et al., 2012).

### Stability of Apple Juice

For the untreated apple juice, the light transmittances after heating or freeze–thaw treatment were higher than that before treatment. Sediments also occurred in untreated apple juice after heating or freeze–thaw treatment (Table 1). No change in light transmittance and no sediment were observed in the apple juice clarified by magnetic immobilized laccase under alternating magnetic field combined with ultrafiltration or microfiltration. The same stability was also observed for the clarifications by free or immobilized laccase under magnetic stirring combined with ultrafiltration. However, the clarification by immobilized laccase under magnetic stirring combined with microfiltration created an unstable apple juice, which can be due to more soluble polymers remained in apple juice after microfiltration, resulting in sediments after heating or freeze–thaw treatment.

**TABLE 1:** Stability of apple juice after heating or freezing-thaw treatment.

**Apple juice**	**T (%)**	**70°C, 30 min**		**Freeze-thaw**	
		**T (%)**	**Sediment**	**T (%)**	**Sediment**
Untreated	68.1 ± 2.1^a^	73.3 ± 1.9^b^	Yes	74.1 ± 1.5^b^	Yes
AMF + MIL + UF	93.5 ± 3.1^a^	93.9 ± 2.2^a^	No	92.7 ± 2.5^a^	No
AMF + MIL + MF	81.9 ± 2.8^a^	82.1 ± 1.8^a^	No	81.0 ± 2.6^a^	No
MS + MIL + UF	82.9 ± 2.3^a^	82.5 ± 2.1^a^	No	83.8 ± 2.6^a^	No
MS + MIL + MF	54.0 ± 1.7^a^	69.2 ± 1.9^b^	Yes	70.3 ± 2.2^b^	Yes
MS + FL + UF	89.3 ± 2.9^a^	89.7 ± 3.1^a^	No	89.1 ± 2.7^a^	No

*T, Light transmittance; AMF, alternating magnetic field; MIL, magnetic immobilized laccase; UF, ultrafiltration; MF, microfiltration; MS, mechanical stirring; FL, free laccase. Means with different superscript lowercase letters within the same line are significantly different (*p* < 0.05).*

## Conclusion

Based on the present research, Cu^2+^-chelated magnetic silica nanoparticles were fabricated and regarded as good carriers for laccase immobilization. Magnetic immobilized laccase exhibited higher reaction rate under alternating magnetic field. The treatment of apple juice with immobilized laccase could combine with microfiltration and provided stable juice with less loss in total phenolic compounds and antioxidant activity. Good operation stability of the enzyme treatment has been proved in MSFB under alternating magnetic field. Application of this combination strategy in large-scale juice clarification and its economic analysis based on pilot data should be investigated in future study.

## Data Availability Statement

The raw data supporting the conclusions of this article will be made available by the authors, without undue reservation.

## Author Contributions

FW: conceptualization, writing, original draft preparation, review, and editing. MO-F: experimental investigation and formal analysis. LX: experimental design, validation, and data curation. ZD: supervision, review, and editing. ZG: methodology. All authors provided approval for the publication of the content.

## Conflict of Interest

The authors declare that the research was conducted in the absence of any commercial or financial relationships that could be construed as a potential conflict of interest.
