# Supplementary material for: Immobilization of Laccase on Magnetic Chelator Nanoparticles for Apple Juice Clarification in Magnetically Stabilized Fluidized Bed
Source: Front Bioeng Biotechnol. 2020 Jul 2;8:589. doi: 10.3389/fbioe.2020.00589 (PMC7343707; doi:10.3389/fbioe.2020.00589)
Supplement: Supplementary file 1 [file Data_Sheet_1.docx]

Supplementary Material

# Supplementary Figures and Tables

## Supplementary Figures


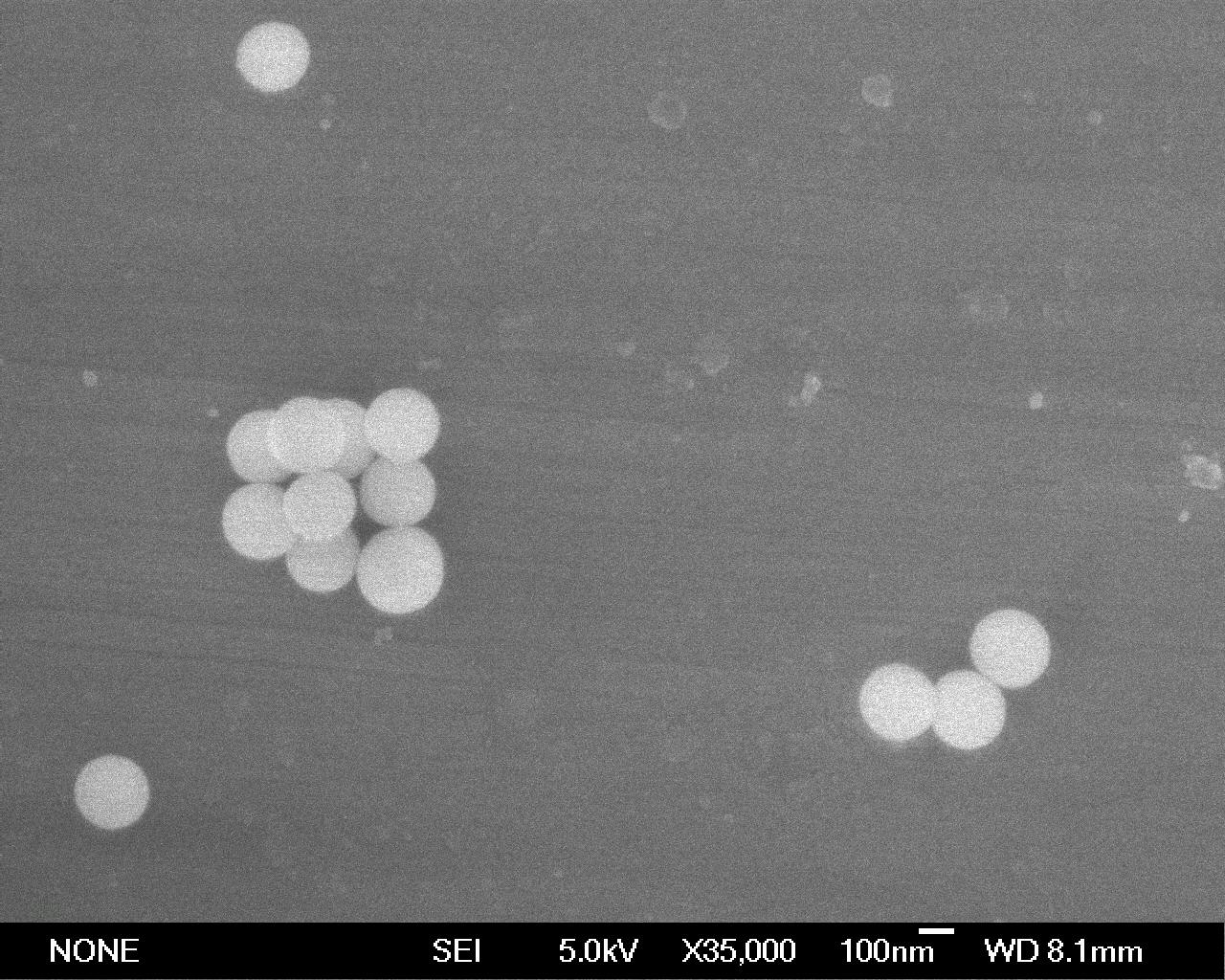


**Supplementary Figure 1.** SEM image of magnetic silica nanoparticles

**Supplementary Figure 2.** Effect of laccase concentration on activity recovery, immobilization efficiency and relative activity of laccase immobilized on Cu^2+^-chelated magnetic silica nanoparticles

(a)

(b)

**Supplementary Figure 3.** Thermal and storage stability of immobilized laccase

(a)

(b)

(c)

**Supplementary Figure 4.** Size distribution of polymer in apple juice after the treatments with magnetic immobilized laccase under different condition: (a) mechanical stirring + free laccase; (b) mechanical stirring + magnetic immobilized laccase; (c) alternating magnetic field + magnetic immobilized laccase

## Supplementary Tables

**Supplementary Table 1.** Kinetic parameters of magnetic immobilized laccase

| Kinetic parameters | Free laccase | Immobilized laccase |
| --- | --- | --- |
| *K_m_* [mM] | 1.22 ± 0.02 ^a^ | 3.17 ± 0.09 ^b^ |
| *kcat* [min^-1^] | 145 ± 5 ^a^ | 151 ± 6 ^a^ |
| *kcat*/*K_m_* [mM^-1^ min^-1^] | 119 ± 4 ^a^ | 48 ± 2 ^b^ |

Means with different superscript lowercase letters within the same line are significantly different (p < 0.05).
